# Supplementary figures and images for: Cerebral blood volume sensitive layer-fMRI in the human auditory cortex at 7T: Challenges and capabilities
Source: PLoS One. 2023 Feb 9;18(2):e0280855. doi: 10.1371/journal.pone.0280855 (PMC9910709; doi:10.1371/journal.pone.0280855)

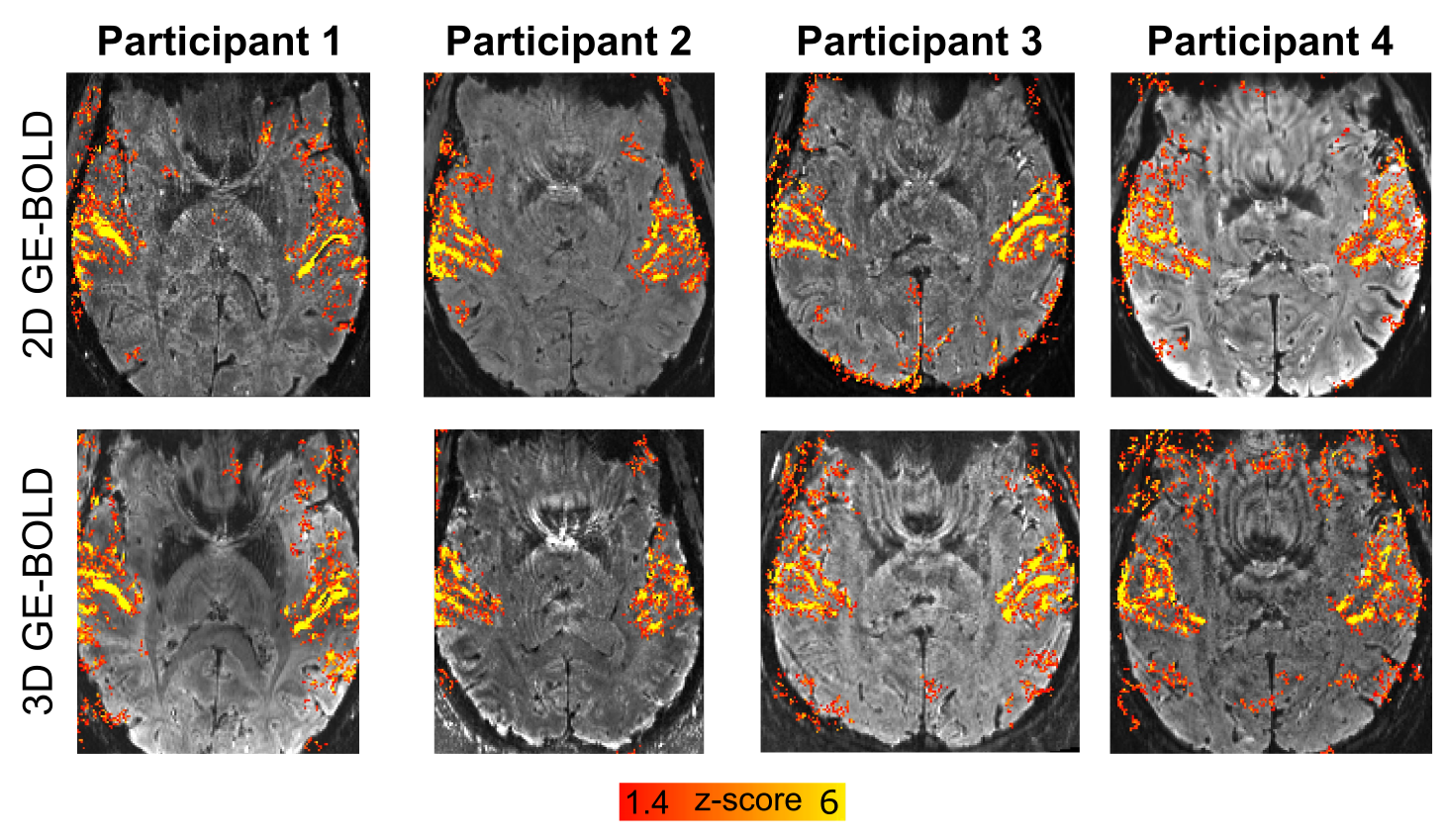

Supplement: S1 Fig — Z-scored activation maps overlayed on distortion corrected mean GE-BOLD EPI images (per participant and readout). The color map was chosen to match the VASO data displayed in Fig 2A. (TIF) [file pone.0280855.s001.tif]

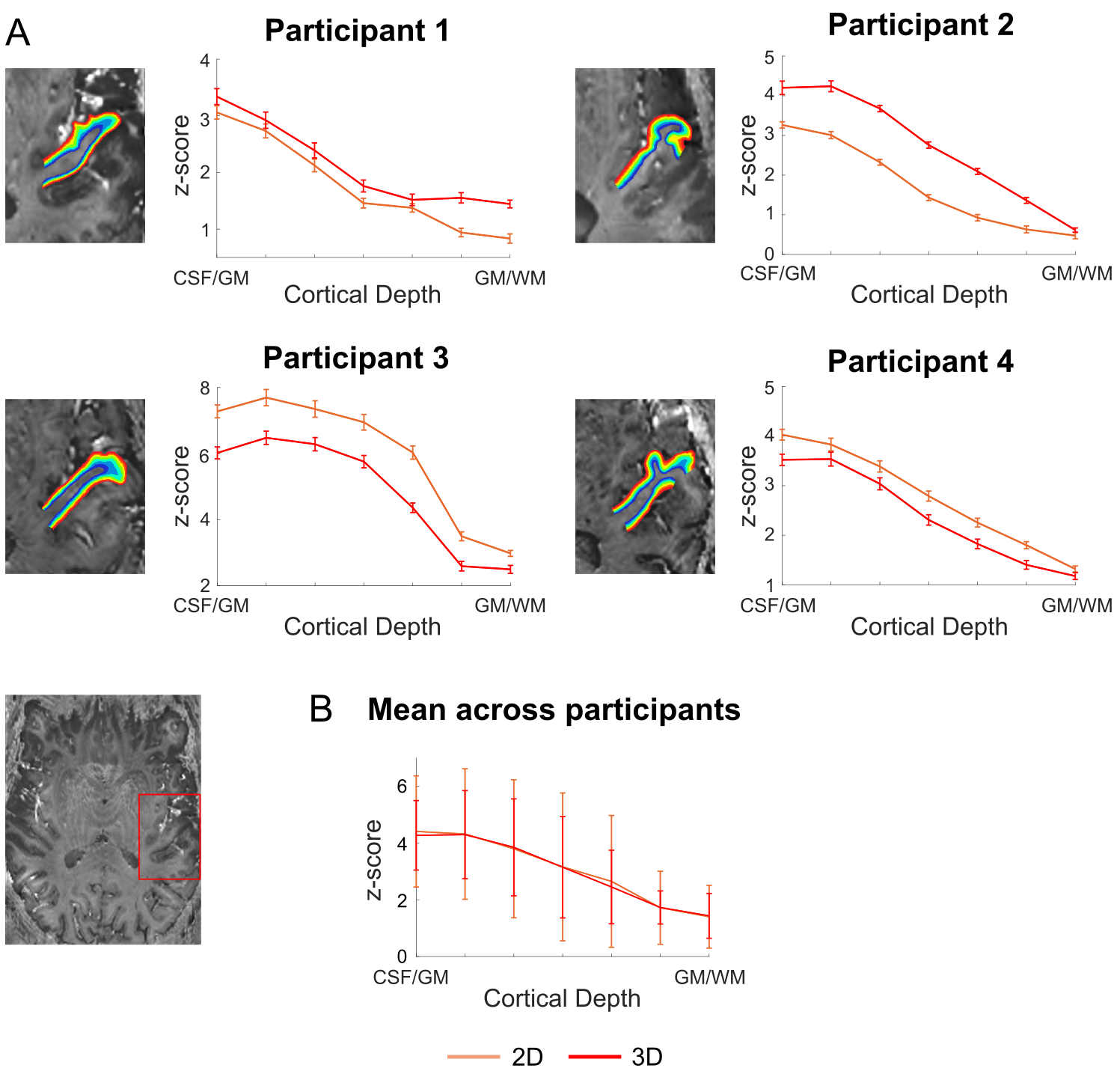

Supplement: S2 Fig — (A) Functional layer-dependent changes across depths for each participant. The BOLD data is coming from the same anatomically-based ROI that was used to calculate the layer-dependent VASO changes in Fig 3. (B) Average z-scored layer-dependent activation changes across participants. (TIF) [file pone.0280855.s002.tif]

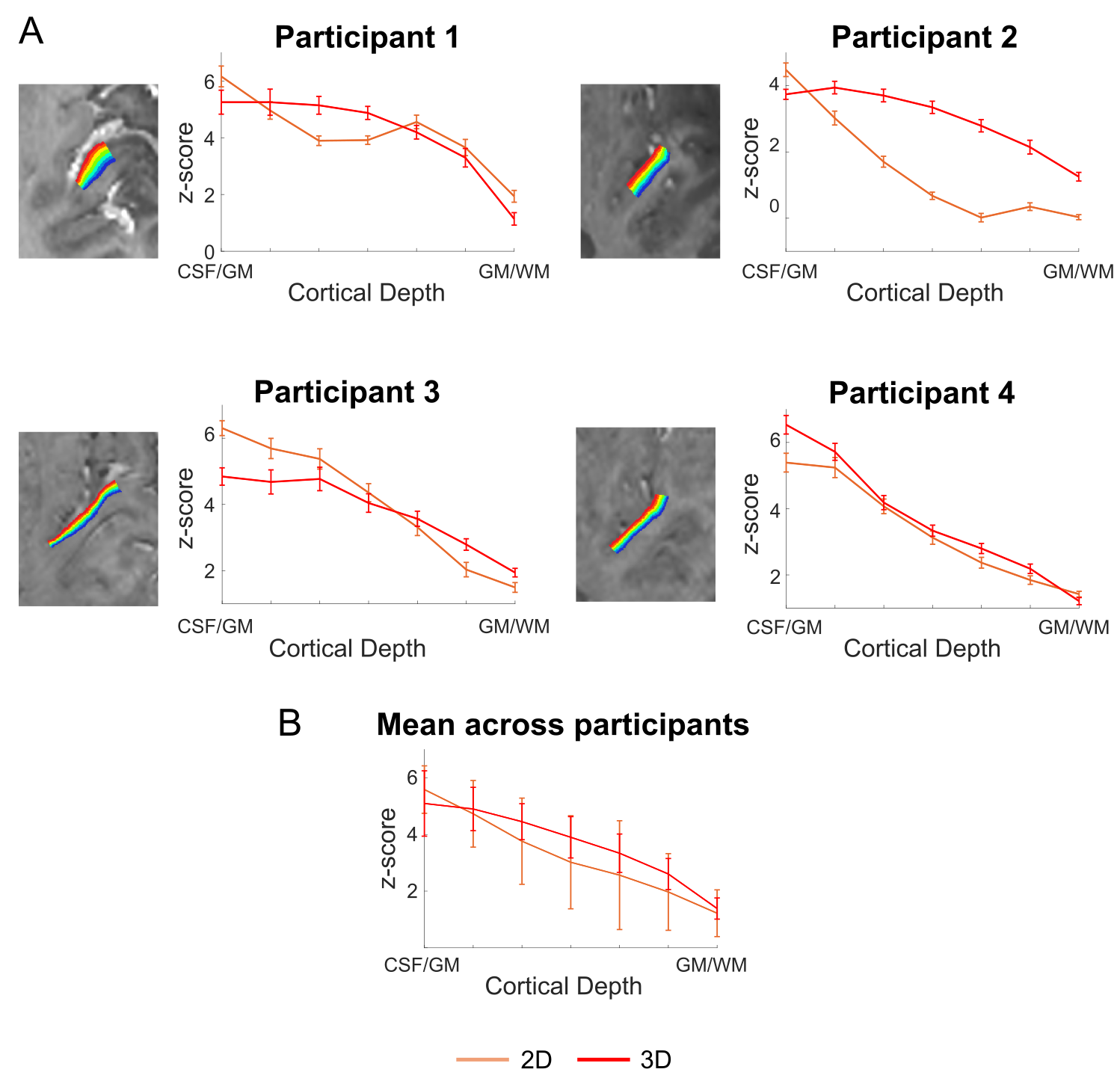

Supplement: S3 Fig — (A) Functional layer-dependent changes across depths for each participant. The BOLD data is coming from the same functional activation-based ROI that was used to calculate the layer-dependent VASO changes in Fig 4, drawn on an axial slice as shown in Fig 3. (B) Average z-scored layer-dependent activation changes across participants. (TIF) [file pone.0280855.s003.tif]

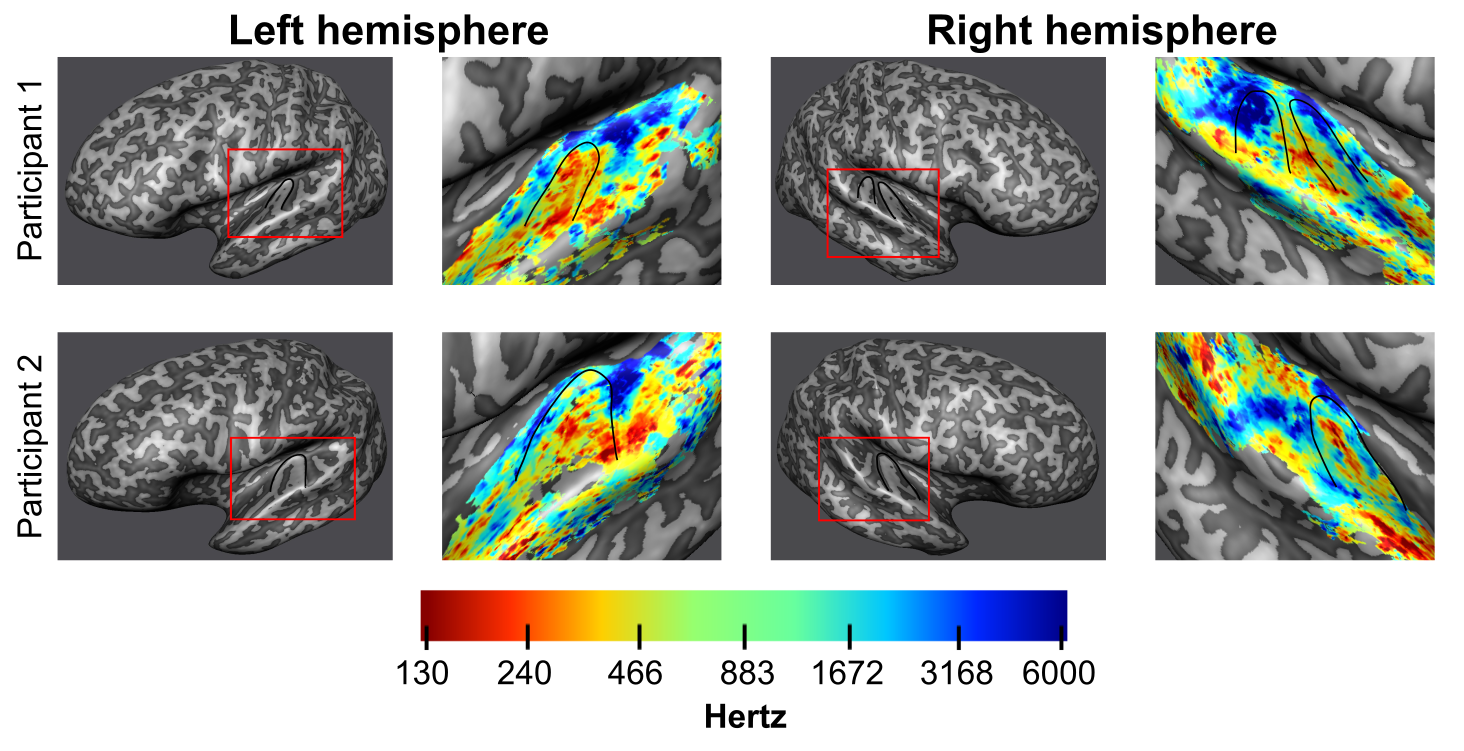

Supplement: S4 Fig — Inflated mid-gray matter surface meshes were created to visualize tonotopic maps created with the BOLD data. On the right of each inflated surface, tonotopic maps are displayed for both hemispheres of the two participants. Heschl’s Gyrus is outlined in black. A tonotopic high-low-high frequency preference gradient is visible in the data. (TIF) [file pone.0280855.s004.tif]
